# Variable-number tandem-repeat markers for typing *Mycobacterium intracellulare *strains isolated in humans

**DOI:** 10.1186/1471-2180-10-93

**Published:** 2010-03-29

**Authors:** Frédéric-Antoine Dauchy, Sébastien Dégrange, Alain Charron, Michel Dupon, Yi Xin, Cécile Bébéar, Jeanne Maugein

**Affiliations:** 1 Service de Maladies Infectieuses et Tropicales, Hôpital Pellegrin, Université Victor Segalen Bordeaux 2, 33076 Bordeaux, France; 2 Laboratoire de Bactériologie EA 3671, Université Victor Segalen Bordeaux 2, CHU de Bordeaux, 33076 Bordeaux Cedex, France

## Abstract

**Background:**

*Mycobacterium intracellulare*, a species of the *Mycobacterium avium complex*, may be the cause of severe lung, lymphatic node, skin and bone/joint infections, as well as bacteriemia. The goal of this work was to identify Mycobacterial Interspersed Repetitive Unit-Variable Number Tandem Repeat (MIRU-VNTR) markers and to study their variability in a collection of isolates of *M. intracellulare *collected in humans. We studied 61 isolates collected in humans between 2001 and 2008, as well as the reference strain, *M. intracellulare *ATCC 13950.

**Results:**

We identified 45 MIRU-VNTR candidates, of which 17 corresponded to the MIRU-VNTR identified in the genome of *M. intracellulare *ATCC 13950. Among the 45 potential MIRU-VNTR, seven were selected for use in a MIRU-VNTR assay applied to our collection of isolates. Forty-four patterns were found by MIRU-VNTR typing and the discriminatory power of the assay was high with a Hunter-Gaston diversity index of 0.98. We do not have evidence of a particular distribution of MIRU-VNTR polymorphism according to clinical situation.

**Conclusions:**

Our results suggest that MIRU-VNTR typing could be used for molecular epidemiological studies applied to *M. intracellulare*.

## Background

Due to their genetic and phenotypic diversity, epidemiological and pathological studies of non-tuberculous mycobacteria are complex. These bacteria are difficult to eradicate because of their natural resistance to the antibiotics frequently used against tuberculosis. Because of their saprophytic and ubiquitous nature, the diagnosis of non-tuberculous mycobacterial disease depends on criteria provided by the American Thoracic Society (ATS) [1].

*Mycobacterium intracellulare *belongs to the *Mycobacterium avium *complex, and has an important role in pathology. In humans, *M. intracellulare *may be the cause of severe lung, lymphatic node, skin and bone/joint infections, as well as bacteriemia [2]. The presence of an immunodepressing context, like that caused by HIV/AIDS, constitutes a risk factor for the *M. avium *infection, but not for the *M. intracellulare *infection. *M. intracellulare *is more frequently isolated at infection stages, as defined by the ATS, than is *M. avium *[3,4]. Most available methods to identify and differentiate strains of *M. intracellulare *are difficult and have limited discriminatory power. The PCR-RFLP method has been used for the typing of *M. avium *[5]. The repeated sequences of VNTR (Variable-Number of Tandem-Repeats), and in particular MIRU (Mycobacterial Interspersed Repetitive Units) have been used for the genotyping of several species of non-tuberculous mycobacteria. The full genomes of *M. avium *and *M. paratuberculosis *have been sequenced allowing the description of MIRU-VNTR in these species [6-9]. MIRU-VNTR markers applied to the genetic typing of *M. intracellulare *have been described very recently [10]. The full genome of *M. intracellulare *has not been published yet, but the sequences of 353 contigs from *M. intracellulare *ATCC 13950 have been publicly available since 2008.

The goal of our work was to identify MIRU-VNTR markers from the genome sequence of *M. intracellulare *ATCC 13950 and to study their variation in a collection of 61 *M. intracellulare *isolates collected at infection or colonizing stages, as defined by the ATS, and from pulmonary or extra-pulmonary sites.

## Methods

### Strain collection

Different MIRU-VNTR were studied in a group including 61 *M. intracellulare *isolates collected under colonization (10 isolates) or infection stages (51 isolates) in humans, and the reference strain *M. intracellulare *ATCC 13950, named strain 1 in our study. The 61 clinical isolates were obtained from 51 patients between January 2001 and January 2008. All isolates were collected in the Bacteriology Department of the Bordeaux University Hospital, except for six which came from Brittany, another region of France (isolates 43, 44, 47, 48, 53 and 57). The average age of patients was 68 years, with a range of 5 to 86. The male/female sex ratio of patients was 0.94. Some patients presented concurrent conditions: HIV infection (strains 39 and 41), cystic fibrosis (strains 43, 49, 50, and 51), blood-related cancer (strains 24 and 62), and lung cancer (strains 7 and 12). Several isolates were collected from the same patients at different times, following a relapse of the illness: isolates 9 and 30 in 2006, isolates 13 and 17 in 2002 and 2005, respectively, isolates 16, 19, 40, and 46 between 2005 and 2008, isolates 22 and 60 in 2006, isolates 23 and 61 in 2007, isolates 28 and 42 in 2007, isolates 35 and 36 in 2007 and 2008, respectively, and isolates 37 and 38 in 2002 and 2003, respectively. The pulmonary or extrapulmonary origin of the isolate, presence or absence of an illness meeting the ATS criteria, gender of the patient, place of residence, and year of isolation were recorded. The isolates were cultured on Löwenstein-Jensen medium. Identification was conducted using Gen-probe^® ^(BioMérieux, France) or GenoType^® ^(Hain Lifescience) for *M. avium *and *M. intracellulare*.

The present project is in compliance with the Helsink Declaration (Ethical Principles for Medical Research Involving Human Subjects). Strains were collected from specimen as part of the patients' usual care, without any additional sampling. All patient data shown in the present work were anonymously reported, without offering any possibility to trace the actual patients.

### Preparation of mycobacterial DNA

Mycobacterial DNA was obtained following the method of Baulard *et al. *[11]. A bacterial suspension from a recent culture (< 1 month) was suspended in 500 μL of TE 1× buffer (Tris/HCl pH 8, EDTA) with 1% of Triton. Suspensions were then incubated for 30 min at 90°C in order to inactivate the bacteria. The DNA from the supernatant was directly used as a template.

We then analyzed the *M. intracellulare *isolates using two techniques: (i) PCR-RFLP as described by Picardeau *et al. *and based on amplification of genomic sequences between IS*1311 *and IS*1245 *(5) and (ii) the MIRU-VNTR method using newly identified MIRU-VNTR markers. We used PCR-RFLP as a comparison to the MIRU-VNTR method.

### Identification of MIRU-VNTR markers

MIRU-VNTR were identified from the sequenced genome of the strain *M. avium *104 (GenBank:08595), by using the program Tandem Repeats Finder http://minisatellites.u-psud.fr. A minimum threshold of 80% homology was used and a sequence of 45 or more base-pairs was required in order for it to be clearly identified on an electrophoresis gel. Only the potential MIRU-VNTR not already described [6,7] were retained.

The genome sequence of *M. intracellulare *ATCC 13950 has been effectively available since 2008 in contig format (GenBank no. ABIN01000000). The 353 available contigs were examined sequentially with the goal of identifying potential MIRU-VNTR using the program and the criteria described above.

To screen for variability in the number of MIRU-VNTR loci, PCR primers targeting the regions flanking the loci were designed. As a preliminary step, the different MIRU-VNTR candidates were tested with specific primers to amplify DNA from a set of 9 randomly chosen *M. intracellulare *isolates, as well as the reference strain ATCC 13950.

Each locus was amplified individually and analyzed by conventional agarose gel electrophoresis. To confirm that length polymorphisms were the result of repeat copy number variations, PCR products were purified with the Wizard^® ^PCR preps DNA purification system (Promega) and sequenced using the fluorescence-labeled dideoxynucleotide technology according to the manufacturer's recommendations (Applied Biosystems). Using this approach, seven MIRU-VNTR loci were selected and taken forward for full assessment.

### PCR amplification of MIRU-VNTR

The PCR reaction was composed of 1 U Go *Taq *Flexi DNA polymerase (Promega); 1 μM of each primer; 1 μM dNTP; 5 μL of 5× buffer solution; 1.5 mM of MgCl_2_; 1 μL of dimethyl sulfoxyde (DMSO, Sigma); and 25 μL of distilled H_2_O. The mixture was added to 5 μL of DNA, diluted at a 1/5 ratio. Amplification conditions were as follows: 1 cycle of 5 min at 94°C; 40 cycles of 30 s at 94°C, 30 s at 58°C, and 30 s at 72°C; and 1 cycle of 7 min at 72°C. To detect difference in repeat numbers, the PCR products were analyzed by electrophoresis in a 1% agarose gel.

### MIRU-VNTR stability study

MIRU-VNTR stability was studied on four clinical isolates, chosen randomly, before and after 10 sequential liquid cultures in the Bactec^® ^MGIT medium (Becton-Dickinson Microbiology Systems). DNA was extracted and subjected to PCR amplification.

### Data analysis

An allele number string, based on the number of repeats at each locus, was assigned to all isolates. The number of repeated motifs was rounded to the next highest number, as previously described [6]. As such, the number of repeated sequences equaling zero signified that the PCR product corresponded to the surrounding area only, without the MIRU-VNTR motif. The discriminatory power of combined MIRU-VNTR loci was calculated using the Hunter-Gaston discriminatory index (HGDI) [12]. Genetic diversity was assessed by allelic diversity (h) [13].

Phylogenetic relationships between the different isolates were analyzed using the program Bionumerics^® ^v.5.0 (Applied Maths). Two different techniques were used to represent the relationships between isolates, (i) A phenogram using phenetic UPGMA methods. (ii) A minimum spanning tree. The minimum spanning tree was generated in order to visualize the relationships between a large number of isolates in a single compact image. Complexes were created if neighbors differed by no more than two of the seven alleles. Each clonal complex is distinguished from its neighbors by an elongation greater than or equal to three loci. The minimum spanning tree was created based on the categorical coefficient and the priority rule 'highest number of single-locus variants'. A detailed description of analysis using minimum spanning tree can be found in the study by Schouls *et al. *[14].

## Results

### Identification of MIRUs

Four [6] and eight MIRU-VNTR [7] have been already described for *M. avium *and *M. paratuberculosis*, respectively. Because of the phylogenetic similarity between these species and *M. intracellulare*, it was predicted that several of these MIRU-VNTR could also be used in typing *M. intracellulare *isolates [15]. Thus we included these MIRU-VNTR loci, named MIRU 1 through 4 (Bull *et al.*), MIRU 32, 292, X3, 25, 3, 7, 10, and 47 (Thibault *et al.*), in our study. The analysis of the genome of *M. avium *104 identified 120 Tandem Repeat (TR) sequences of which 16 were selected based on their degree of homology and their size (MIN 1 through MIN 16). Examination of the 353 contigs of *M. intracellulare *ATCC 13950 identified 310 TRs of which 17 were used in the study (MIN 17 through MIN 33). Thus a total of 45 TR loci were studied.

The polymorphism of the selected 45 TR loci was initially investigated on a set of nine randomly chosen isolates of *M. intracellulare *(isolates 2 through 10), as well as the reference strain *M. intracellulare *ATCC 13950 (strain 1). Thirty-four MIRU-VNTR were absent during amplification of one or more isolates while four MIRU-VNTR did not demonstrate polymorphism on the isolates tested, they were thus eliminated. One of the 12 MIRU-VNTR already described for *M. avium *and *M. intracellulare*, MIRU 3 (Bull at al.), was polymorphic with different allele sizes. None of the new MIRU-VNTR identified from the strain *M. avium *104 could be validated on our set of 10 isolates of *M. intracellulare*. Consequently, of the 45 candidate MIRU-VNTR only seven, MIRU 3 (Bull *et al.*), MIN 18, MIN 19, MIN 20, MIN 22, MIN 31, and MIN 33, were present and exhibited polymorphism.

The stability of the seven polymorphic MIRU-VNTR markers was studied on four isolates. No difference was seen in the gel profiles before and after 10 passages in MGIT medium. Thus, an MIRU-VNTR scheme was proposed, including seven markers. It allowed unambiguous type assignment using agarose gels, with PCR products ranging in size between 200 and 750 bp. Characteristics of each MIRU-VNTR marker are shown in Table 1. As the genome sequence of *M. intracellulare *was available only in a contig format and was not annotated, it was impossible to determine where the MIRU-VNTR were located in the genome. The sizes of the unit repeat ranged from 53 to 57 bp. Sequencing of the different size PCR products at each of the seven loci from each of the 10 isolates confirmed the sizes and sequences of individual MIRU-VNTR loci.

**Table 1:** MIRU-VNTR characteristics

	Oligonucleotide primers								
				
	Forward primer			Reverse primer			Repeat size (bp)	Size of the amplicons (range, bp)	%^a^
3 (Bull *et al.*)	5'-ACATTCACCCTGTCCATTC-3'			5'-CCTCCTTACGGAGCAGGAA-3'			53	200-350	-
MIN 18	5'-GCCGAACCATTTGGCGAAC-3'			5'-GGATTCGGCCGCGCAATTC-3'			56	200-500	98
MIN 19	5'-CATGGTTCGCCCTCTACAC-3'			5'-TAGGGGCAGGTCATCGAAG-3'			53	200-380	98
MIN 20	5'-GCTGAGCTACAGCCTCGAC-3'			5'-CGACGCCGATGACGTAAAC-3'			55	320-620	98
MIN 22	5'-TCAGGAATGGGTCCGGTTC-3'			5'-AGCTCGTGACGACGGAAAC-3'			57	200-450	98
MIN 31	5'-CGACCGCATCCAGAAACAG-3'			5'-GCTCTATGACGACCTCAAG-3'			57	280-420	95
MIN 33	5'-GTGCAGTTCAACCACGAAC-3'			5'-GGCGTTGAACACGTTGGTG-3'			54	350-750	95

^a ^% identity percentage between Tandem-Repeats

### Typing of clinical isolates

The PCR-RFLP method and the set of seven MIRU-VNTR were used to type a collection of 62 *M. intracellulare *isolates. Specimens were cultured from the respiratory tract (51 isolates) or from extra-pulmonary sites (10 isolates + reference strain ATCC) and represented infection (51 isolates + reference strain ATCC) or colonization (10 isolates) stages, respectively.

PCR-RFLP did not provide the expected discriminating power for the 62 *M. intracellulare *isolates. We obtained polymorphic and complex patterns, containing up to 15 bands. Because of these weak and complex amplifications, we were not able to accurately type the panel of isolates. Nevertheless, we were able to confirm the identity of strains sequentially collected from the same patients. Thus, the PCR-RFLP method seems to be accurate to compare close isolates of *M. intracellulare*. PCR-RFLP reported by Picardeau *et al. *might be useful for *M. avium *but not *M. intracellulare *typing.

The seven MIRU-VNTR were amplified very efficiently in all 62 isolates and the size variations of the amplicons were an exact multiple of repeats. Results are shown in Table 2. Analysis of the combination of the seven MIRU-VNTR loci for the 62 *M. intracellulare *isolates revealed 44 MIRU-VNTR types. Strains isolated at different times from the same patient following a relapse of the illness showed identical MIRU-VNTR allele profiles. Marker MIN 33 was the most discriminating MIRU-VNTR, displaying seven different alleles with repeat copy numbers equal to zero or ranging from 2 to 7 depending on the isolate. Marker MIN 31 was the most homogeneous marker, most of the isolates harboring 2 or 3 repeat units of 57 bp. This was also reflected by the discriminatory power estimated by the HGDI, calculated on the 52 non epidemiologically linked isolates. Only the first isolate from each patient was included in this analysis. The most discriminant marker MIN 33 had a HGDI of 0.85 while the less discriminant one, MIN 31, had a HGDI of 0.60. The overall discriminatory index of the MIRU-VNTR method was 0.98.

**Table 2:** MIRU-VNTR allelic distribution and allelic diversity, among 52 independent *M. intracellulare *isolates.

	Number of isolates with the specified MIRU-VNTR copy number								
		
	0	1	2	3	4	5	6	7	allelic diversity (h)
MIRU 3 (Bull *et al.*)	9	13	17	13* ^a^					0.74
MIN 18	10	1	19	7	15*				0.72
MIN 19	22	1		21	8*				0.63
MIN 20	1	19	2	7	19	4*			0.71
MIN 22	3	2	24	10	13*				0.68
MIN 31	5	3	15	29*					0.59
MIN 33	4		6	5	12*	6	11	8	0.83

^a ^Asterisk denotes the profile of the reference strain ATCC 13950.

**Figure 1:**
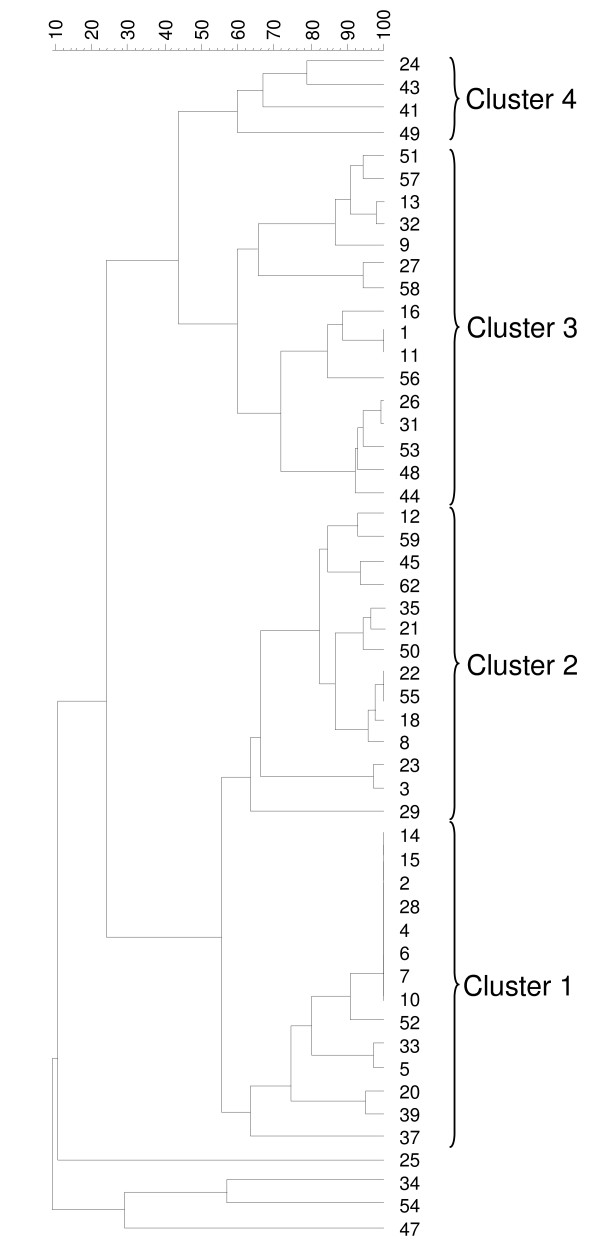
**UPGMA tree of the MIRU-VNTR types for the 52 independent *M. intracellulare *isolates**. 1: ATCC strain. 2-62: clinical isolates.

**Figure 2:**
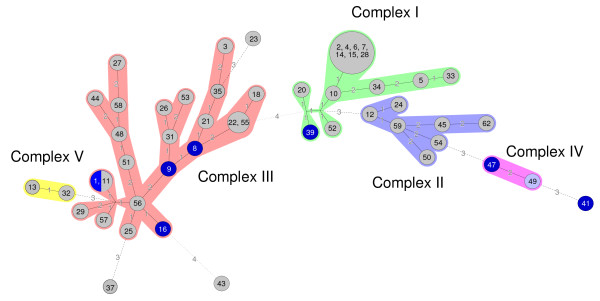
**Minimum spanning tree of the MIRU-VNTR types for the 52 independent *M. intracellulare *isolates**. Each circle denotes a particular MIRU-VNTR type with the isolates corresponding to this genotype indicated by numbers (1, ATCC strain, 2-62, clinical isolates). Size of circles differs according to the number of isolates. The distance between neighboring genotypes is expressed as the number of allelic changes and is indicated by numbers. Surrounding colors correspond to clonal complexes. Grey circles correspond to isolates of pulmonary sources and blue circles to isolates of extra-pulmonary sources.

As a complementary analysis, the MIRU-VNTR profiles were imported into Bionumerics^® ^(Applied-maths), and the genetic relationships of the 52 independant isolates were deduced by the construction of an UPGMA tree (figure 1) and a minimum spanning tree (figure 2). The minimum spanning tree allowed us to distinguish five clonal complexes, of which three were predominant (shown as three separate colors encircling the isolates in figure 2). Complex I was composed of 14 isolates, with a principal group of seven isolates. Since the origin and collection dates were known, we could eliminate the chance of laboratory contamination and the presence of a communal source. The reference strain was identical to clinical isolate number 11 and is located in complex III. The UPGMA tree allowed us to distinguish four clusters (figure 1). The isolates belonging to the clonal complex I are found in cluster 1, except for isolate 34 which is unclustered. Most of the clonal complex II strains are found in cluster 2 except for strain 24 (cluster 4) and strain 54 (not clustered). The clonal complex III isolates are all situated in clusters 2 and 3. There was no obvious link between the MIRU-VNTR typing and the clinical situation, the year when the isolates were collected, the patient age, the geographical origin or the origin site.

## Discussion

We described seven MIRU-VNTR markers, applicable in the typing of *M. intracellulare*. We studied 61 isolates, collected from 51 patients between 2001 and 2008, as well as the reference strain *M. intracellulare *ATCC 13950.

The MIRU-VNTR technique was conducted using different candidate MIRU-VNTR chosen from the genome of *M. avium *and from *M. intracellulare *contigs. Out of 45 candidate MIRU-VNTR studied, only seven were retained, of which six came from *M. intracellulare *contigs. Among the 17 MIRU-VNTR from contigs, 11 had to be eliminated due to inadequate amplification. The primers found to be ineffective on the study strains were also ineffective on the reference strain. The lack of reference strain amplification could be related to differences between the sequenced genome and the genome of the reference strain in our possession.

The MIRU-VNTR technique provides numerous advantages: it provides a rapid, adaptable technique to comment on the presence of clonal complexes within isolates linked using an epidemiological method [16]. Coding the results as a series of numbers allows an easy exchange of results between different labs. On the practical side, this technique also enables evaluation of the possibility of laboratory contamination in cultures from different isolates. Using MIRU-VNTR markers, we also confirmed the identity of isolates collected from the same patients when they had a relapse of their illness. This stability was observed *invitro *with subcultures of the same isolate, and *invivo *for the same infected patient. This result contrasted with results obtained by the MIRU-VNTR technique on strains of *M. tuberculosis*, which provided an example of frequent exogenous infections [17]. We did not find any difference in the genetic profile of serial strains found in our patients, which permitted us to exclude the possibility of re-infection with a new strain of *M. intracellulare*.

For the clustering analysis of MIRU-VNTR profiles, a graphing algorithm termed minimum spanning tree was used. This method has been introduced by some authors to improve analysis of VNTR profiles [14]. Similar to maximum-parsimony phylogenetic tree reconstruction methods, minimum spanning tree constructs a tree that connects all the genetic profiles in such a way that the summed genetic distance of all branches is minimized. The differences in mathematical approach between minimum spanning tree and UPGMA methods explain changes in strains clustering. Thus, minimum spanning tree allowed us to group strains which were unclustered with UPGMA (isolates 54 in complex II and 34 in complex I).

Our study permitted us to characterize the statistical power of the MIRU-VNTR technique as applied to *M. intracellulare*. The global discriminatory index of 0.98 presented in this work confirmed the possible use of this technique, in agreement with results obtained with other species of the avium complex [7]. Interestingly, Ichikawa *et al. *[10] also described an HGDI of 0.98 for the MLVA of *M. intracellulare*. Forty-four MIRU-VNTR types were obtained in our study for the 61 *M. intracellulare *clinical isolates, a number similar to that described by Ichikawa [10]. Our results confirmed the data very recently described for *M. intracellulare *[10] showing that this method seemed to harbor a great discriminatory power for identification of genetically similar isolates.

*Mycobacterium avium-intracellulare *complex agents, in addition to a broad host range, are environmental mycobacteria found in numerous biotopes including the soil, water, aerosols, and vegetation. Nevertheless, little is known about genetic variations among patient and environmental isolates of *M. intracellulare*. Development of genotyping methods could allow further studies to examine the possible reservoirs of this pathogen in the environment, their diversity and their role as sources of infection in humans.

MIRU-VNTR are present in diverse metabolic or regulatory systems, as part of synthesis or degradation of lipids, nucleic acids, proteins, energy production, or signal transduction [18]. For our part, because we did not have access to the genome of *M. intracellulare *other than in contig format, we were not able to measure the location of the MIRU-VNTR in inter- or intragenic regions. In this study, we did not have evidence of a particular distribution of MIRU-VNTR polymorphism according to clinical situation. To date, publications on the virulence of non-tuberculous mycobacteria are preliminary. Genotyping using the MIRU-VNTR technique could offer the opportunity for better classification of strains, and could be used for to research on virulence mechanisms in non-tuberculous mycobacteria.

## Conclusions

This study allowed us to describe seven MIRU-VNTR markers, applicable in the typing of *M. intracellulare*. The loci in this MIRU-VNTR assay were highly discriminating and stable over time. MIRU-VNTR typing could be used for molecular epidemiological studies of *M. intracellulare *strains. Furthermore, data obtained by MLVA could be shared in a web database for *M. intracellulare*, as has already been done for other bacterial species.

## Authors' contributions

FAD participated in the design of the study, carried out the molecular studies, participated in the phylogenetic analysis and draft the manuscript. SD and AC participated in the molecular studies and the phylogenetic analysis. MD participated in the design of the study. YX participated in the molecular studies. CB participated in the design of the study and to draft the manuscript, JM conceived the study, and participated in its design and coordination, and helped to draft the manuscript. All the authors read and approved the final manuscript.
